# Community composition, bacterial symbionts, antibacterial and antioxidant activities of honeybee-associated fungi

**DOI:** 10.1186/s12866-022-02580-4

**Published:** 2022-06-27

**Authors:** Pu Cui, Kun Kong, Yong Yao, Zhongdi Huang, Shuping Shi, Peng Liu, Yechen Huang, Naeem Abbas, Linsheng Yu, Yinglao Zhang

**Affiliations:** 1 School of Life Sciences, Anhui Agricultural University, Hefei, 230036 China; 2 School of Plant Protection, Anhui Agricultural University, Hefei, 230036 China; 3 Laboratory of Functions and Applications of Medicinal Plants, Guizhou Medical University, Guiyang, 550014 China

**Keywords:** *Apis mellifera ligustica*, Honeybee-associated fungi, Bacterial symbionts, Antibacterial activity, Antioxidant activity, Secondary metabolites

## Abstract

**Background:**

Fungi associated with insects represent one potentially rich source for the discovery of novel metabolites. However, a comprehensive understanding of the fungal communities of *Apis mellifera ligustica* remains elusive.

**Results:**

Here, we investigated the phylogenetic diversity and community composition of honeybee-associated fungi using combination of culture-dependent and culture-independent approaches. A total of forty-five fungi were isolated and purified from the *Apis mellifera ligustica*, royal jelly, and honeycomb, which belonged to four classes and eleven different genera. Furthermore, 28 bacterial 16S rRNA gene sequences were obtained by PCR from the fungal metagenome. High-throughput sequencing analyses revealed that the fungal communities were more diverse, a total of 62 fungal genera were detected in the honeybee gut by culture-independent method, whereas only 4 genera were isolated by culture-dependent method. Similarly, 247 fungal genera were detected in the honeycomb, whereas only 4 genera were isolated. In addition, we assessed the antibacterial and antioxidant activities of fungal isolates. Most fungal crude extracts obtained from the cultivation supernatant exhibited antioxidant activities. Only two fungal crude extracts displayed moderate activity against *Escherichia coli* and *Staphylococcus aureus*. Chemical analysis of *Chaetomium subaffine* MFFC22 led to the discovery of three known compounds, including cochliodinol (**1**), emodin (**2**), chrysophanol (**3**). Among them, cochliodinol (**1**) showed intense DPPH radical scavenging activity with the 50% inhibitory concentration (IC_50_) of 3.06 μg/mL, which was comparable to that of the positive ascorbic acid (IC_50_ = 2.25 μg/mL). Compound **2** displayed weak inhibitory activities against *Micrococcus tetragenus* and *S. aureus*.

**Conclusions:**

This research provided a fundamental clue for the complex interactions among honeybees, fungi, bacterial symbionts, and the effects on the honeybee. Furthermore, the diversity of honeybee-associated fungi had great potential in finding the resource of new species and antioxidants.

**Supplementary Information:**

The online version contains supplementary material available at 10.1186/s12866-022-02580-4.

## Background

Insects are the most abundant group of animals on the Earth. Over one million species of insects have been named and widely distributed in various habitats [1, 2]. The great diversity of insects nurtures a large microbial community association with the insect. Some microbial species were found valuable functions in nutrition and protection in social insects, such as termites, ants, and bees [3–5]. It is worth mentioning that the relationship between insects and their associated fungi capture researchers' attention, for instance, beetle-fungus farming symbiosis, fungus-cultivating termites, and fungus-farming ants [6–8]. In addition, reports indicate that some bacteria were present in fungal hyphae [9, 10]. It is worth considering to evaluate in many further aspects of the associations between fungi and insects as well as fungi and bacteria.

Insect-associated fungi are vital microbial sources of natural bioactive products. The insect-associated fungi isolated from arthropod cadavers, leaf-cutting ants, and stoneflies were contained new microbial species [11–13]. Insect-associated fungi could enhance insects' fitness by producing bioactive compounds [14]. Furthermore, many novel compounds have been discovered from insect-associated fungi, and these compounds had the potential as immune inhibitors, antibacterial agents, and biofungicides [15–17]. However, the current research in metabolites produced by insect-associated fungi is still not comprehensive.

As a social insect, honeybees belong to the class Insecta and the order Hymenoptera in the phylum Arthropoda. It is a critical species for agricultural production as pollinators [18]. Various parts of honeybee larvae and adults, their food, and honeycomb harbor numerous microorganisms, which play a significant role in food digestion, pollination, and antagonistic effect against different pathogens [19]. Especially, fungi associated with honeybee may provide material for pollen degradation or assist in royal jelly maturation, and also can be a food source [20, 21]. For example, the Brazilian stingless bee larva grows by eating fungal mycelia of *Monascus* inside the brood cell [22]. Therefore, honeybees are a potential model of fungus-host-symbiont interactions, which is worth exploring. As a high royal jelly-producing honeybee, *Apis mellifera ligustica* acquired fungi from their diet, the surrounding environment, or mates [23]. However, only a few reports involve fungi isolated from specimens associated with *A. mellifera ligustica* [24]. Our knowledge about the species, biological activity, and secondary metabolites of these associated fungi are limited until now. Here, we investigated the diversity of fungi from *A. mellifera ligustica* using culture-dependent and culture-independent methods and explored the bacterial symbionts, biological activity, and secondary metabolites of the fungi isolated from *A. mellifera ligustica* (larvae, adults), honeycomb, and royal jelly.

## Results

### Identification of cultivable fungi associated with honeybee

**Fig. 1:**
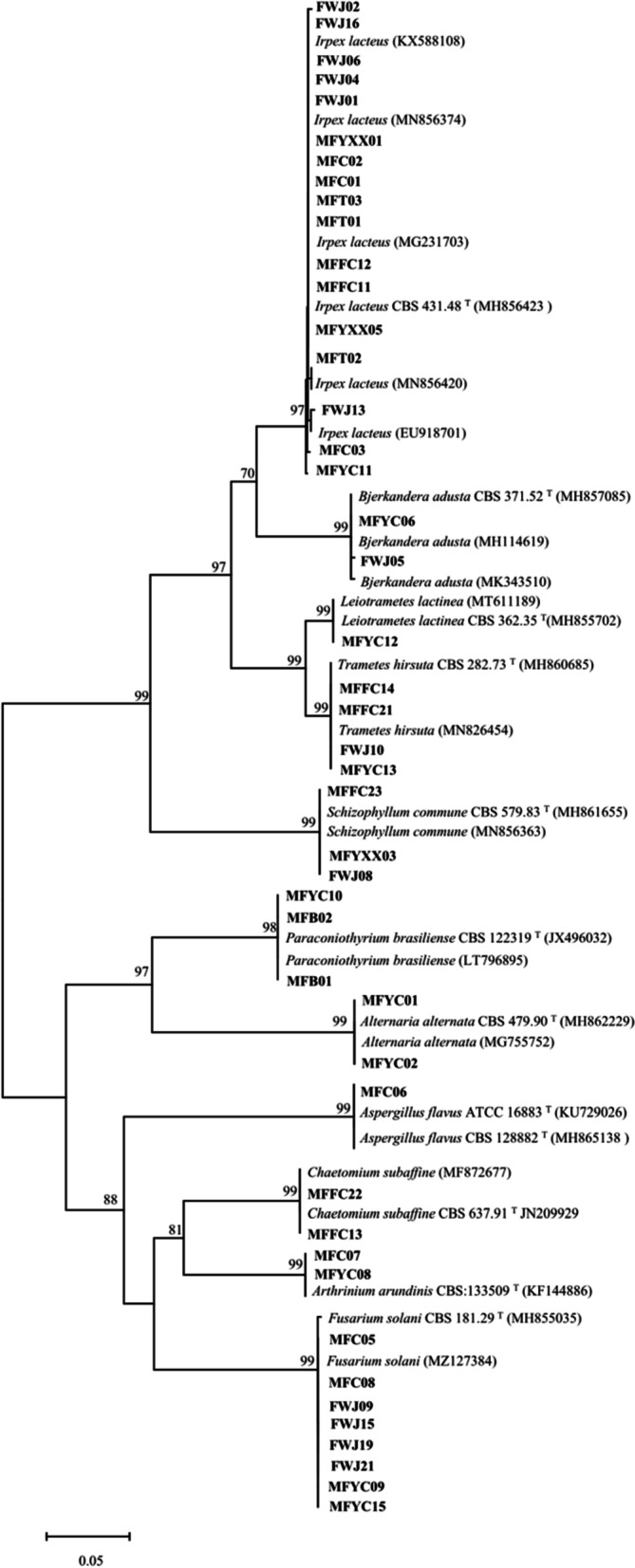
Neighbor-joining phylogenetic tree of ITS gene sequences of honeybee-associated fungi. The numbers at the nodes represent bootstrap support, based on a neighbor-joining analysis of 1000 replicates. Only the bootstrap value odes represent bootstrap sbranches.The GeneBank taxa are designated by species name with accession number while our isolates are designated by code name

**Fig. 2:**
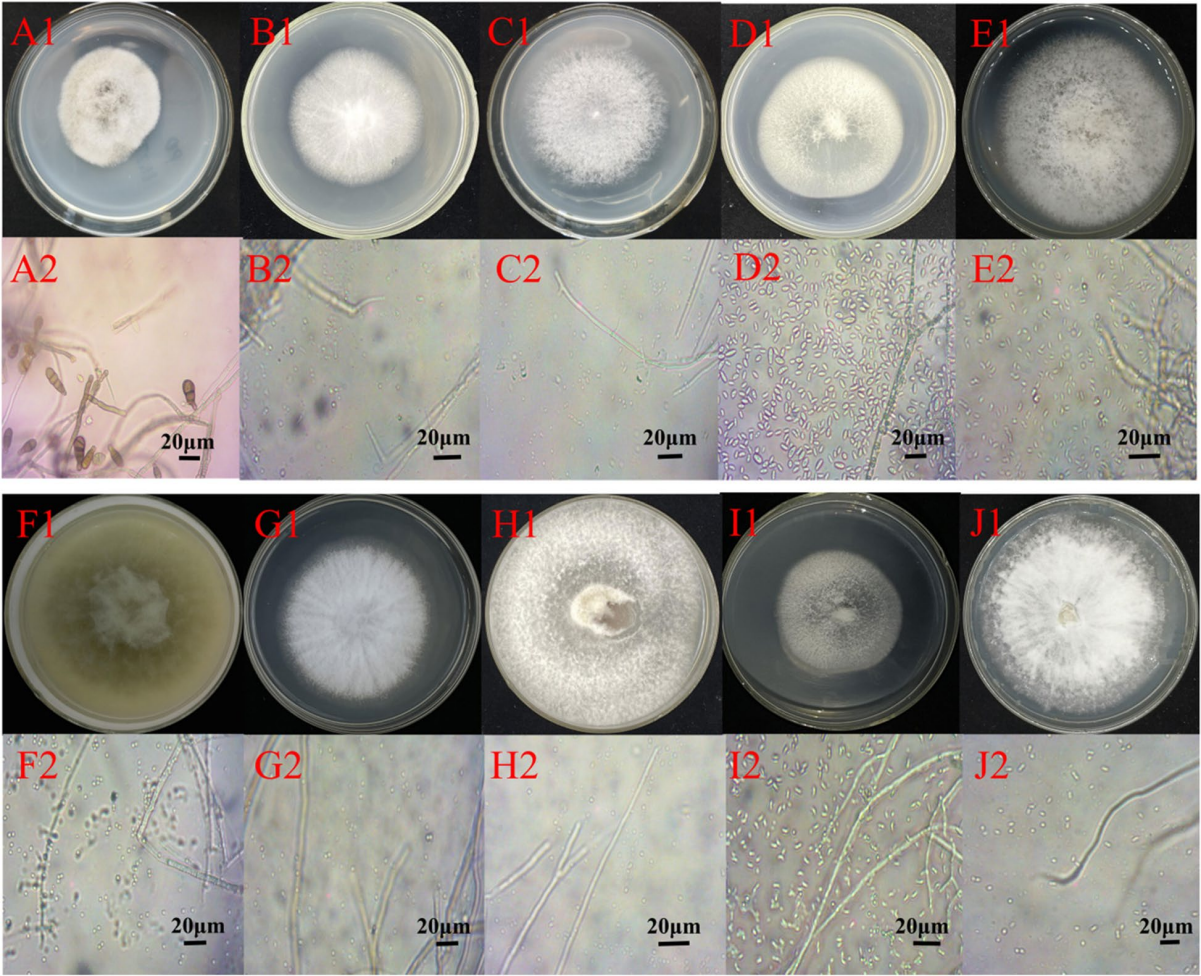
The morphological and microscopic characteristics of some honeybee- associated fungi. Colony (A1) and conidia (A2) of *Alternaria alternata* MFYC01; colony (B1) and conidia (B2) of *Paraconiothyrium brasiliense* MFB02; colony (C1) and conidia (C2) of *Irpex lacteus* MFT03; colony (D1) and conidia (D2) of *Fusarium solani* FWJ19; colony (E1) and conidia (E2) of *Arthrinium arundinis* MFYC08; colony (F1) and conidia (F2) of *Chaetomium subaffine* MFFC22; colony (G1) and conidia (G2) of *Trametes hirsuta* FWJ10; colony (H1) and conidia (H2) of *Bjerkandera adusta* MFYC06; colony (I1) and conidia (I2) of *Schizophyllum commune* FWJ08; colony (J1) and conidia (J2) of *Leiotrametes lactinea* MFYC12

In this study, a total of forty-five fungi were isolated from the honeycomb, royal jelly, larvae, and different parts of honeybee on nine different media (Table 1, Fig. 1). The morphological and microscopic characteristics of the ten representative strains are shown in Fig. 2. Among them, seven strains were isolated from honeycomb, thirteen from royal jelly, ten from larvae, two from honeybee cuticle, seven from gut, three from head, and three from hypopharyngeal gland. ITS sequence analysis of the forty-five isolates revealed that the fungal isolates belonged to two different phyla (Basidiomycota and Ascomycota) and eleven different genera. Twenty-seven isolates (60.0%) were distributed in the Agaricomycetes within the phylum Basidiomycota. The other eighteen isolates were grouped into three classes [Sordariomycetes (26.7%), Dothideomycetes (11.1%), and Eurotiomycetes (2.2%)] within Ascomycota.

**Table 1:** Phylogenetic analysis of cultivable fungi associated with *Apis mellifera ligustica*, and similarity values for ITS gene sequences

Isolate code	Host	part	Closest match	Accession number of closest match (Genbank) (Genbank)	Proposed identity	Coverage/Max ident	GenBank accession number
MFFC14	Honeycomb		*Trametes hirsuta*	MN826454	*Trametes hirsuta*	96/99	OK184580
MFFC21	Honeycomb		*Trametes hirsuta*	MN826454	*Trametes hirsuta*	94/99	OK184581
FWJ10	Royal jelly		*Trametes hirsuta*	MN826454	*Trametes hirsuta*	98/99	OK184569
MFYC13	Honeybee	Larvae	*Trametes hirsuta*	MN826454	*Trametes hirsuta*	88/99	OK184590
MFFC11	Honeycomb		*Irpex lacteus*	MG231703	*Irpex lacteus*	96/99	OK184577
MFFC12	Honeycomb		*Irpex lacteus*	MG231703	*Irpex lacteus*	97/99	OK184578
MFT01	Honeybee	Head	*Irpex lacteus*	MN856374	*Irpex lacteus*	98/98	OK184584
MFT02	Honeybee	Head	*Irpex lacteus*	MN856420	*Irpex lacteus*	97/99	OK184585
MFT03	Honeybee	Head	*Irpex lacteus*	MN856374	*Irpex lacteus*	98/99	OK184586
MFC01	Honeybee	Gut	*Irpex lacteus*	MG231703	*Irpex lacteus*	96/99	OK184571
MFC02	Honeybee	Gut	*Irpex lacteus*	MG231703	*Irpex lacteus*	95/99	OK184601
MFC03	Honeybee	Gut	*Irpex lacteus*	MN856374	*Irpex lacteus*	95/98	OK184572
MFYXX01	Honeybee	Hypopharyngeal gland	*Irpex lacteus*	MN856374	*Irpex lacteus*	99/99	OK184591
MFYXX05	Honeybee	Hypopharyngeal gland	*Irpex lacteus*	MG231703	*Irpex lacteus*	98/98	OK184593
FWJ01	Royal jelly		*Irpex lacteus*	MN856374	*Irpex lacteus*	94/99	OK184563
FWJ02	Royal jelly		*Irpex lacteus*	MG231703	*Irpex lacteus*	96/99	OK184564
FWJ04	Royal jelly		*Irpex lacteus*	MN856374	*Irpex lacteus*	95/99	OK184565
FWJ06	Royal jelly		*Irpex lacteus*	KX588108	*Irpex lacteus*	91/100	OK184567
FWJ13	Royal jelly		*Irpex lacteus*	EU918701	*Irpex lacteus*	95/98	OK184595
FWJ16	Royal jelly		*Irpex lacteus*	MG231703	*Irpex lacteus*	89/98	OK184570
MFYC11	Honeybee	Larvae	*Irpex lacteus*	MN856420	*Irpex lacteus*	97/98	OK184588
MFYC06	Honeybee	Larvae	*Bjerkandera adusta*	MH114619	*Bjerkandera adusta*	96/99	OK184587
FWJ05	Royal jelly		*Bjerkandera adusta*	MK343510	*Bjerkandera adusta*	95/99	OK184566
MFYC12	Honeybee	Larvae	*Leiotrametes lactinea*	MT611189	*Leiotrametes lactinea*	97/99	OK184589
MFFC23	Honeycomb		*Schizophyllum commune*	MN856363	*Schizophyllum commune*	96/99	OK184583
MFYXX03	Honeybee	Hypopharyngeal gland	*Schizophyllum commune*	MN856363	*Schizophyllum commune*	95/100	OK184592
FWJ08	Royal jelly		*Schizophyllum commune*	MN856363	*Schizophyllum commune*	96/99	OK184568
MFFC13	Honeycomb		*Chaetomium subaffine*	MF872677	*Chaetomium subaffine*	94/100	OK184579
MFFC22	Honeycomb		*Chaetomium subaffine*	MF872677	*Chaetomium subaffine*	89/100	OK184582
MFC05	Honeybee	Gut	*Fusarium solani*	MZ127384	*Fusarium solani*	92/99	OK184573
MFC08	Honeybee	Gut	*Fusarium solani*	MZ127384	*Fusarium solani*	96/99	OK184576
FWJ09	Royal jelly		*Fusarium solani*	MZ127384	*Fusarium solani*	92/99	OK184594
FWJ15	Royal jelly		*Fusarium solani*	MZ127384	*Fusarium solani*	93/99	OK184596
FWJ19	Royal jelly		*Fusarium solani*	MZ127384	*Fusarium solani*	91/99	OK184597
FWJ21	Royal jelly		*Fusarium solani*	MZ127384	*Fusarium solani*	92/99	OK184598
MFYC09	Honeybee	Larvae	*Fusarium solani*	MZ127384	*Fusarium solani*	93/99	OK184605
MFYC15	Honeybee	Larvae	*Fusarium solani*	MZ127384	*Fusarium solani*	91/100	OK184606
MFYC01	Honeybee	Larvae	*Alternaria alternata*	MG755752	*Alternaria alternata*	95/99	OK184602
MFYC02	Honeybee	Larvae	*Alternaria alternata*	MG755752	*Alternaria alternata*	96/99	OK184603
MFC06	Honeybee	Gut	*Aspergillus flavus*	JQ070072	*Aspergillus flavus*	96/99	OK184574
MFB01	Honeybee	Cuticle	*Paraconiothyrium brasiliense*	LT796895	*Paraconiothyrium brasiliense*	95/99	OK184599
MFB02	Honeybee	Cuticle	*Paraconiothyrium brasiliense*	LT796895	*Paraconiothyrium brasiliense*	97/99	OK184600
MFYC10	Honeybee	Larvae	*Paraconiothyrium brasiliense*	LT796895	*Paraconiothyrium brasiliense*	100/99	OK285068
MFC07	Honeybee	Gut	*Arthrinium arundinis*	KF144886	*Arthrinium arundinis*	90/100	OK184575
MFYC08	Honeybee	Larvae	*Arthrinium arundinis*	KF144886	*Arthrinium arundinis*	90/100	OK184604

Fungal isolates belonging to class Agaricomycetes were assigned to two orders, including the Agaricales (3 isolates) and Polyporales (24 isolates). The three strains in Agaricales were isolated from the honeycomb, royal jelly, and hypopharyngeal gland, respectively, which showed highly similar to *Schizophyllum commune* with more than 99% identity. The three strains in Agaricales were isolated from the honeycomb, royal jelly, and hypopharyngeal gland, respectively, which showed highly similar to *Schizophyllum commune* with an identity of more than 99%. The strains of Polyporales were isolated from most of the samples and represented by the family Polyporaceae, Irpicaceae and Phanerochaetaceae. Among them, most strains (17 isolates) belonging to genus *Irpex* were identified as *Irpex lacteus.* Notably, the strain FWJ13 showed only 98% similarity to *I. lacteus*. In addition, two strains isolated from honeybee larvae were identified as *Bjerkandera adusta* and *Leiotrametes lactinea*, respectively. Moreover, *B. adusta* was also found in royal jelly. The last four strains belonging to *Trametes* genus showed similar to *Trametes hirsuta* with an identity of 99%.

The other representative class was Sordariomycetes, including three orders (Hypocreales, Sordariales, and Xylariales). Eight isolates belonging to the family Hyporcreaceae exhibited a sequence match of more than 99% to *Fusarium solani*. *Fusarium* spp. were found in association with honeybee gut, larvae, and royal jelly. Both strains belonging to Sordariales were isolated from the honeycomb, identified as *Chaetomium subaffine* with an identity of 100%. The other two strains belonging to the Xylariales were identified as *Arthrinium arundinis*, which were found to be most related to honeybee intestines and larvae.

The five fungal sequences of Dothideomycetes were grouped into Pleosporales, including *Paraconiothyrium brasiliense* (3 isolates), and *Alternaria alternate* (2 isolates). The *P. brasiliense* with an identity of 99% were isolated from honeybee cuticle and larvae, respectively. The genus *Alternaria* was only isolated from honeybee larvae.

Finally, only one isolate belonging to class Eurotiomycetes was grouped into the order Eurotiales, which belonged to the genus *Aspergillus* with a high sequence match to *Aspergillus flavus* (> 99%). The strain was isolated from honeybee gut.

### Culture-independent community

**Fig. 3:**
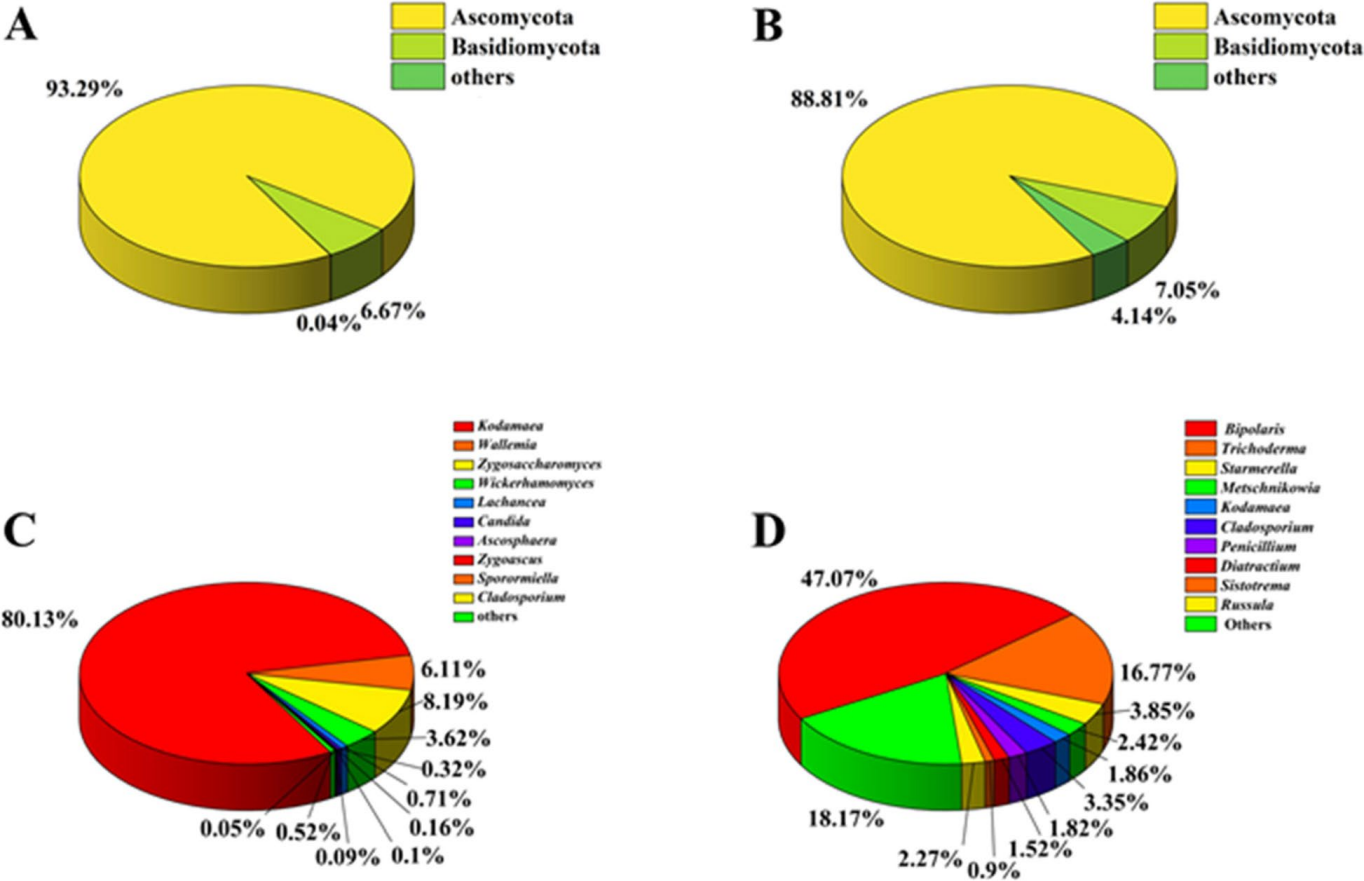
Analysis of culture-independent microbial communities. Relative abundance of OTUs at phylum (A) and genus (C) level of honeybee gut; relative abundance of OTUs at phylum (B) and genus (D) level of honeycomb. 290,634, 117,478 high quality reads were generated from honeybee gut and honeycomb

The ITS1 region was performed to analyze the fungal community within the honeybee gut and honeycomb by using Illumina Miseq sequencing. A total of 408,112 high-quality fungal clean reads were generated from two populations with 3 replicates for each population (honeybee gut: MFF1_1, MFF1_2, MFF1_3, honeycomb: FC_01, FC_02, FC_03) (Supplementary Table S1 and S2). A total of 3 fungal phyla were identified in honeybee gut samples, including Ascomycota, Basidiomycota, and unclassified fungi, the average abundance was 93.29, 6.67, 0.04%, respectively (Fig. 3A). Ascomycota (88.81% of the average abundance) and Basidiomycota (7.05%) were identified in honeycomb samples (Fig. 3C). Regarding the composition of fungal community, all samples were dominated at the phylum level by Ascomycota. The proportions of Ascomycota in the honeybee gut were higher than in the honeycomb. The community composition was further analyzed at the genus level. 62, 247 genera were identified across investigated samples of honeybee gut and honeycomb, respectively. Among them, *Kodamaea* (80.13%), *Zygosaccharomyces* (8.19%), *Wallemia* (6.11%), and *Wickerhamomyces* (3.62%) had higher abundance in the honeybee gut (Fig. 3B). However, *Bipolaris* (47.07%), *Metschnikowia* (18.17%), *Starmerella* (3.85%), *Trichoderma* (16.77%), *Kodamaea* (3.35%) had higher abundance in the honeycomb (Fig. 3D). The relative abundance of yeast such as *Kodamaea*, *Zygosaccharomyces*, and *Wickerhamomyces* was higher in the honeybee gut than in the honeycomb. There are differences in fungal communities of honeybee gut and honeycomb samples between the two approaches. Culture-independent community analysis showed more diverse fungal communities than did the culture-dependent method.

### Identification of bacterial symbionts

In this study, a total of twenty-eight bacterial symbionts 16S rRNA were observed from genomic DNA extractions of the honeybee-associated fungi (Table2). Among them, nineteen, six, two, and one strain were isolated from the host of the Agaricomycetes, Sordariomycetes, Dothideomycetes, and Eurotiomycetes, respectively. All bacterial symbionts were divided into four genera by initial BLAST comparisons in GenBank and were putatively identified as *Bacillus velezensis*, *Bacillus siamensis*, *Microbacterium tumbae*, *Pandoraea sputorum*, and *Achromobacter xylosoxidans*. These bacterial symbionts were mainly identified as *B. velezensis* and *B. siamensis* in Bacillaceae. Moreover, *B. velezensis* was obtained from fungi of different honeybee samples or parts, such as honeybee gut, hypopharyngeal gland, head, cuticle, larvae, honeycomb, and royal jelly.

**Table 2:** The taxonomic classification of bacterial symbionts from cultivable fungi associated with *Apis mellifera ligustica*, and similarity values for 16S rRNA gene sequences

**Isolate code**	**Closest match**	**Accession number of closest match (Genbank)**	**Proposed identity**	**Coverage/Max ident**	**GenBank accession number**
MFFC11x	*Bacillus velezensis*	NR075005	*Bacillus velezensis*	99/99	OK147634
MFFC23x	*Bacillus velezensis*	NR075005	*Bacillus velezensis*	98/99	OK147635
MFB01x	*Bacillus velezensis*	NR075005	*Bacillus velezensis*	99/99	OK147627
MFC01x	*Bacillus velezensis*	NR116240	*Bacillus velezensis*	98/99	OK147628
MFC02x	*Bacillus velezensis*	NR075005	*Bacillus velezensis*	99/99	OK147629
MFC03x	*Bacillus velezensis*	NR075005	*Bacillus velezensis*	98/99	OK147630
MFT03x	*Bacillus velezensis*	NR075005	*Bacillus velezensis*	99/99	OK169608
MFYXX01x	*Bacillus velezensis*	NR075005	*Bacillus velezensis*	98/99	OK147644
MFYXX05x	*Bacillus velezensis*	NR075005	*Bacillus velezensis*	98/99	OK147645
FWJ01x	*Bacillus velezensis*	NR075005	*Bacillus velezensis*	98/99	OK169609
FWJ04x	*Bacillus velezensis*	NR075005	*Bacillus velezensis*	98/99	OK169610
FWJ05x	*Bacillus velezensis*	NR075005	*Bacillus velezensis*	98/99	OK169611
FWJ08x	*Bacillus velezensis*	NR075005	*Bacillus velezensis*	99/99	OK147622
FWJ10x	*Bacillus velezensis*	NR075005	*Bacillus velezensis*	99/99	OK147623
FWJ16x	*Bacillus velezensis*	NR075005	*Bacillus velezensis*	98/99	OK147624
MFYC01x	*Bacillus velezensis*	NR075005	*Bacillus velezensis*	99/99	OK147637
MFYC06x	*Bacillus velezensis*	NR075005	*Bacillus velezensis*	98/99	OK147638
MFYC11x	*Bacillus velezensis*	NR075005	*Bacillus velezensis*	99/99	OK147640
MFYC12x	*Bacillus velezensis*	NR075005	*Bacillus velezensis*	99/99	OK147641
MFYC13x	*Bacillus velezensis*	NR075005	*Bacillus velezensis*	99/99	OK147642
MFT01x	*Bacillus siamensis*	NR117274	*Bacillus siamensis*	100/99	OK147636
MFC06x	*Microbacterium tumbae*	NR156954	*Microbacterium tumbae*	99/98	OK147633
MFC05x	*Pandoraea sputorum*	NR028751	*Pandoraea sputorum*	99/99	OK147631
MFC08x	*Pandoraea sputorum*	NR028751	*Pandoraea sputorum*	99/99	OK147632
FWJ15x	*Pandoraea sputorum*	NR028751	*Pandoraea sputorum*	98/99	OK147624
FWJ21x	*Pandoraea sputorum*	NR028751	*Pandoraea sputorum*	98/99	OK147626
MFYC09x	*Pandoraea sputorum*	NR028751	*Pandoraea sputorum*	98/99	OK147639
MFYC15x	*Achromobacter xylosoxidans*	NR113733	*Achromobacter xylosoxidans*	99/99	OK147643

### Antimicrobial activities

The filter paper disc method was used to evaluate the antibacterial activities of 36 fungal crude extracts, which were obtained from the cultivation supernatant (Table3). Most of the fungal crude extracts showed no antibacterial activities. However, *I. lacteus* FWJ16 displayed moderate activity against *E. coli* with a disc diameter of inhibition zone diameter (IZD) of 10.13 mm, which was weaker than the positive gentamicin sulfate with the IZD of 26.33 mm. Additionally, *P. brasiliense* MFYC10 also displayed moderate activity against *S. aureus* with the IZD of 9.67 mm, which was weaker than the positive gentamicin sulfate with the IZD of 23.92 mm.

**Table 3:** Antimicrobial and antioxidant activities of fungal extracts associated with *Apis mellifera ligustica*

**Proposed identity**	**Isolate code**	***M. tetragenus*** (mm)	***E. coli*** (mm)	S.S.S.S.S.S.S.S.***aureus*** (mm)	**DPPH radical(%)**
*Trametes hirsuta*	MFFC14	NI	NI	NI	NI
*Trametes hirsuta*	MFFC21	NI	NI	NI	12.4 ± 1.8
*Trametes hirsuta*	FWJ10	NI	NI	NI	14.3 ± 3.8
*Trametes hirsuta*	MFYC13	NI	NI	NI	14.4 ± 2.1
*Irpex lacteus*	MFT01	NI	NI	NI	10.3 ± 2.1
*Irpex lacteus*	MFT02	NI	NI	NI	3.6 ± 1.5
*Irpex lacteus*	MFT03	NI	NI	NI	13.7 ± 4.2
*Irpex lacteus*	MFC01	NI	NI	NI	22.6 ± 0.4
*Irpex lacteus*	MFC03	NI	NI	NI	12.0 ± 1.3
*Irpex lacteus*	MFYXX01	NI	NI	NI	5.5 ± 3.7
*Irpex lacteus*	FWJ01	NI	NI	NI	NI
*Irpex lacteus*	FWJ04	NI	NI	NI	14.6 ± 2.0
*Irpex lacteus*	FWJ16	NI	10.13 ± 0.38	NI	12.4 ± 1.8
*Irpex lacteus*	MFYC11	NI	NI	NI	11.2 ± 1.3
*Irpex lacteus*	MFFC11	NI	NI	NI	14.7 ± 2.1
*Irpex lacteus*	MFFC12	NI	NI	NI	15.6 ± 0.6
*Irpex lacteus*	MFC02	NI	NI	NI	14.6 ± 2.0
*Irpex lacteus*	MFYXX05	NI	NI	NI	18.1 ± 2.2
*Irpex lacteus*	FWJ02	NI	NI	NI	8.8 ± 2.3
*Irpex lacteus*	FWJ06	NI	NI	NI	17.2 ± 3.9
*Irpex lacteus*	FWJ13	NI	NI	NI	12.3 ± 2.1
*Bjerkandera adusta*	MFYC06	NI	NI	NI	11.2 ± 1.3
*Bjerkandera adusta*	FWJ05	NI	NI	NI	14.7 ± 2.1
*Leiotrametes lactinea*	MFYC12	NI	NI	NI	26.8 ± 1.0
*Schizophyllum commune*	MFFC23	NI	NI	NI	NI
*Schizophyllum commune*	MFYXX03	NI	NI	NI	NI
*Schizophyllum commune*	FWJ08	NI	NI	NI	7.3 ± 2.2
*Chaetomium subaffine*	MFFC13	NI	NI	NI	82.2 ± 0.8
*Chaetomium subaffine*	MFFC22	NI	NI	NI	91.6 ± 0.4
*Alternaria alternata*	MFYC01	NI	NI	NI	75.5 ± 4.7
*Alternaria alternata*	MFYC02	NI	NI	NI	46.9 ± 1.6
*Aspergillus flavus*	MFC06	NI	NI	NI	40.6 ± 4.6
*Paraconiothyrium brasiliense*	MFB01	NI	NI	NI	9.3 ± 1.2
*Paraconiothyrium brasiliense*	MFB02	NI	NI	NI	83.7 ± 0.3
*Paraconiothyrium brasiliense*	MFYC10	NI	NI	9.67 ± 1.12	74.8 ± 2.8
*Arthrinium arundinis*	MFYC08	NI	NI	NI	44.4 ± 2.2
Gentamicin sulfate ^a^		35.33 ± 0.82	26.33 ± 1.23	23.92 ± 0.12	
Ascorbic acid (Vc) ^b^					97.0 ± 2.6

^a^Gentamicin sulfate as the positive control of pathogenic bacteria; results are presented as the mean ± standard; “NI” means not inhibited; the concentration for the test is 90 µg/filter paper

^b^Ascorbic acid (Vc) as the positive control of DPPH free radical scavenging activity; results are presented as the mean ± standard; “NI” means no activity; the concentration for the test is 166.67 µg/Ml

### Antioxidant activities

**Fig. 4:**
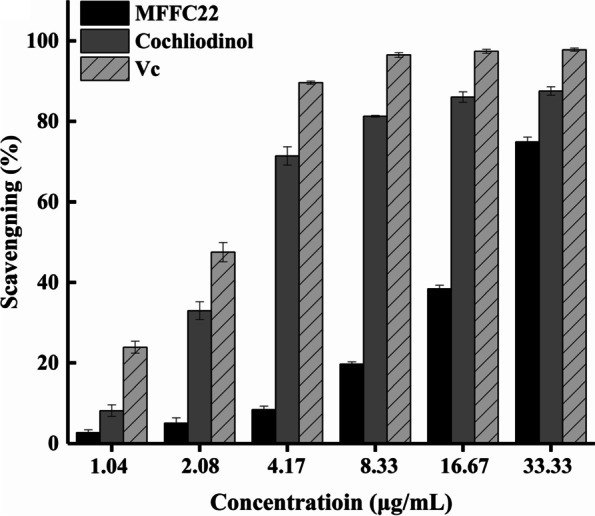
DPPH radical scavenging capacity of cochliodinol (**1**), MFFC22 and ascorbic acid (Vc)

The antioxidant activities of 36 fungal crude extracts are shown in Table3. The results revealed that 32 extracts (88.9%) exhibited antioxidant activities under the concentration of 166.67 µg/mL. Among them, scavenging rates between 10–40%, 40–70%, and 70–100% were found in 19 (52.8%), 3 (8.3%), and 5 (13.9%) strains, respectively. Especially, crude extract of MFFC22 showed the strongest antioxidant activity with the DPPH scavenging activity of 91.6%. Thus, the MFFC22 was further selected to evaluate antioxidant activity at different concentrations (Fig.4). The result showed that the MFFC22 had a higher activity with the increasing concentration. Its IC_50_ on DPPH scavenging activity was 22.11 µg/mL, which was relatively weaker than a commercial natural antioxidant ascorbic acid (Vc, 2.25 µg/mL).

### Identification of the secondary metabolites from *C. subaffine* MFFC22

**Fig. 5:**
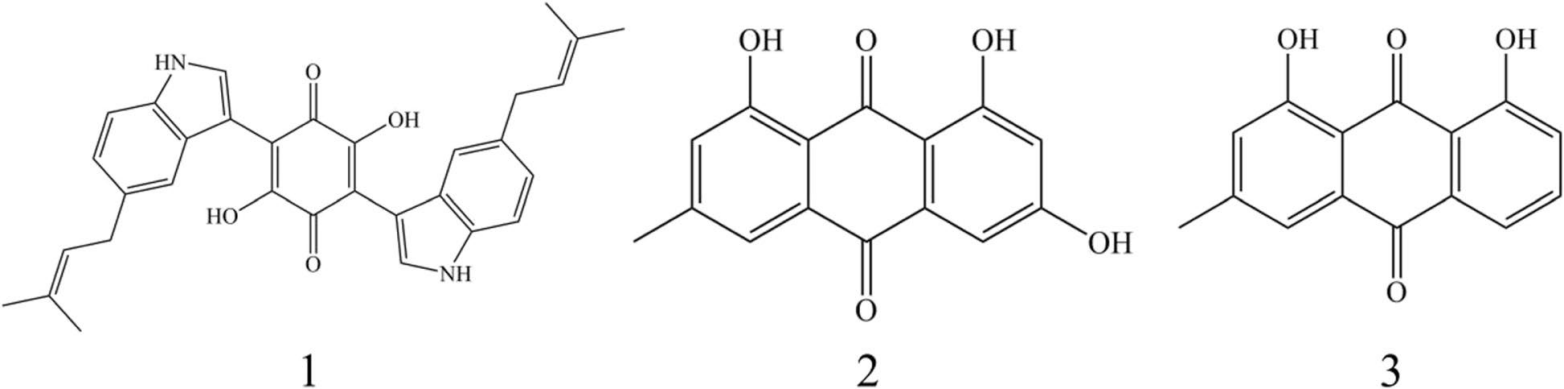
The structure of compounds **1**, **2**, and **3**

Three compounds were purified from the liquid fermentation product of *C. subaffine* MFFC22 and their structures were determined to be cochliodinol (**1**) [25], emodin (**2**) [26], and chrysophanol (**3**) [27] (Fig. 5) by spectroscopic data analyses and comparison of their data in the literature.

Cochliodinol (**1**): purple solid; HR-ESI–MS: m/z: 505.2109 [M—H]^−^, calculated for C_32_H_30_N_2_O_4_ 505.2128; ^1^H NMR (600 MHz, CDCl_3_) *δ*: 8.42 (1H, s, 2,5-OH), 8.14 (1H, s, 1´-NH), 7.60 (1H, s, H2´), 7.44 (1H, s, H4´), 7.34 (1H, d 8.3, H7´),7.09 (1H, d 8.3, H6´), 5.40 (1H, m, H11´), 3.48 (1H, d 7.1, H10´), 1.76 (3H, s, 13´-CH_3_), 1.74 (1H, s, 14´-CH_3_); ^13^C NMR (150 MHz, CDCl_3_): 134.3 s (C8´), 133.7 s (C5´), 131.6 s (C12´), 127.1 d(C2´), 126.2 s (C9´), 124.4 d (C11´), 123.4 d (C6´), 120.8 s (C4´),111.0 d (C7´), 110.7 s (C3, C6), 104.2 s (C3´), 34.6 t (C10´), 25.6 q (C14´), 17.8 q (C13´). Due to the resonance of the benzoquinone ring, the carbonyls resonances (C1, C2, C4, and C5) of cochliodinol were not observed; these carbons can only be observed in THF-d_8_ or CDCl_3_ at -75 ℃ [28].

Emodin (**2**): orange crystals; HR-ESI–MS: m/z: 269.0444 [M—H]^−^, calculated for C_15_H_9_O_5_, 269.0450; ^1^H NMR (600 MHz, acetone-*d*_*6*_) *δ*:12.21 (1H, s, 1-OH), 12.08 (1H, s, 8-OH), 10.15 (1H, s, 3-OH), 7.59 (1H, d 1.5, H5), 7.28 (1H, d 1.5, H4), 7.16 (1H, d 0.6, H7), 6.68 (1H, d 2.0, H2), 2.48 (3H, s, 6-CH_3_); ^13^C NMR (150 MHz, acetone-*d*_*6*_): 191.6 (C9), 182.1(C10), 166.5 (C1), 166.3 (C3), 163.3 (C8), 148.8 (C6), 136.9 (C12), 134.5 (C14), 125.0 (C7), 121.7 (C5), 113.8 (C13), 109.8 (C11), 109.7 (C4), 109.0 (C2), 22.2 (CH_3_).

Chrysophanol (**3**): orange crystals; HR-ESI–MS: m/z: 253.0521 [M—H]^−^, calculated for C_15_H_9_O_4_, 253.0501; ^1^H NMR (600 MHz, CDCl_3_) *δ*: 12.12 (1H, s, 1-OH), 12.02 (1H, s, 8-OH), 7.83 (1H, d 7.2, H5), 7.68 (1H, m, H6), 7.66 (1H, s, H4), 7.30 (1H, d 8.4, H7), 7.11(1H, s, H2), 2.47 (3H, s, 3-CH_3_).

### Antibacterial and antioxidant activities of compounds

The antibacterial activities of the compounds isolated from MFFC22 were shown in Table 4. Specifically, compound **2** displayed weak inhibitory activities against *M. tetragenus* and *S. aureus* with the IZD of 7.00 and 7.33 mm, respectively. Compound **1** had no inhibition effect on three bacterial strains.

**Table 4:** Antibacterial activities of compounds isolated from MFFC22

Compounds	*M. tetragenus* (mm)	*E. coli* (mm)	*S. aureus* (mm)
**1**	NI	NI	NI
**2**	7.00 ± 0.00	NI	7.33 ± 0.47
Gentamicin sulfate^a^	26.00 ± 0.00	20.00 ± 0.00	19.00 ± 0.00

^a^Gentamicin sulfate as the positive control of pathogenic bacteria; results are presented as the mean ± standard; “NI” means not inhibited; the concentration for the test is 30 µg/filter paper

The antioxidant activities of compound **1** isolated from MFFC22 are shown in Fig. 4. Notably, the 50% inhibitory concentration (IC_50_) of cochliodinol (**1**) on DPPH scavenging activity was 3.06 μg/mL, which was comparable to that of positive ascorbic acid (2.25 μg/mL).

## Discussion

Microbial community is extensive among insects. As a special kind of microbial community, insect-associated fungi have been a source of new microbial resources [29, 30] and biologically active natural products [16, 31, 32]. While fungi were present in *A. mellifera ligustica*, the leading research mainly focused on the honeycomb, honeybee gut, pollen, and bread [23, 33–36]. To the best of our knowledge, this is the first report on the diversity, antibacterial and antioxidant activities of culturable fungi from honeycomb, honeybee product (royal jelly), larvae, and different parts of *A. mellifera ligustica*. Forty-five fungi were isolated and characterized by the dilution-plate method and molecular biological identification. Furthermore, three metabolites were purified and characterized from *C. subaffine* MFFC22. Consequently, honeybee-associated fungi can provide a resource for microbial diversity and natural products.

The same fungus of *I. lacteus* was isolated and identified from honeybee gut, head, hypopharyngeal gland, larvae, and royal jelly in our study. It suggests that the fungus is widely distributed in *A. mellifera ligustica*. Some fungi isolated from honeybee larvae, hypopharyngeal gland, honeycomb, and royal jelly were wood-rotting fungi, such as *T. hirsuta*, *S. commune*, *B. adusta*, and *L. lactinea* [37–39]. Besides, the genus *Arthrinium* isolated from honeybee gut and larvae has been reported as a plant pathogen [40]. Compared with the fungal diversity of other insects, some fungi isolated from honeybee were the same as those of other different insects. For example, *I. lacteus* and *P. brasiliense* were also isolated from termites, and *F. solani* was isolated from beetles [41, 42]. *Aspergillus*, *Alternaria*, *Chaetomium*, *Fusarium*, and *Arthrinium* genera have already been found in the honeybee. For example, *Aspergillus*, *Chaetomium*, *Fusarium*, and *Alternaria* sp. were isolated from honeybee gut, and *Arthrinium* sp. was isolated from pollen [23, 33–35]. However, most of these fungi reported in this study are new for this honeybee. For instance, this is the first report of the isolation of *T. hirsuta* and *S. commune* from both honeycomb and royal jelly, *I. lacteus* from all parts of honeybee samples. Additionally, one rare fungus *P. brasiliense* was found in honeybee cuticle, which was reported previously in the gut of *Acrida cinerea* [43].

A total of 62 fungal genera were discovered in honeybee gut by culture-independent method. Among them, *Kodamaea*, *Zygosaccharomyces*, *Wallemia*, and *Wickerhamomyces* were predominant genera. Previous study based on pyrosequencing of ITS region of honeybee gut samples derived from Korea revealed that *Saccharomyces* and *Zygosaccharomyces* were dominant [20]. However, *Starmerella* and *Hanseniaspora* had high relative abundance in honeybee gut samples collected from Italy and Saudi Arabia [16]. Previous studies on predominant genera were inconsistent with each other, and also differed from our results. Presumably, it was due to the different surrounding environments of the sampling sites. Some genera analyzed by culture-independent approaches were also isolated by culture-dependent method, such as *Aspergillus* in honeybee gut, *Trametes*, and *Chaetomium* in honeycomb. However, culture-independent approaches in general revealed higher microbial composition and diversity compared to culture-dependent method. For example, 247 fungal genera were detected in the honeycomb by culture-independent method, whereas only 4 genera were isolated. Interestingly, as the causative agent of chalkbrood disease in honeybee Reserved literature [44], *Ascosphaera* spp. was detected in honeybee gut and honeycomb by culture-independent approach, but it has not been isolated yet.

The DPPH radical scavenging assay indicated that most crude extracts from honeybee-associated fungi showed antioxidant activities. Notably, the crude extract of *C. subaffine* MFFC22 isolated from honeycomb has shown notable antioxidant activity. *Chaetomium* genus is already known to have strong DPPH scavenging activity, such as *C. nigricolor*, *C. globosum*, *C. cruentum* [26, 44, 45]. Nevertheless, *C. subaffine* and its metabolites have not reported DPPH scavenging activity. The compound **1** from MFFC22 showed strong DPPH scavenging activity with the IC_50_ values of 3.06 μg/mL, comparable to the positive ascorbic acid (Vc). Comparing a commercial antioxidant BHT with IC_50_ of 95.7 μM, Dehghan et al. found that compounds **2** and **3** also exerted moderate antioxidant activities against DPPH with IC_50_ of 271.2 and 297.0 μM, respectively [46]. The phenolic compounds with the free hydroxyl groups were well known for robust antioxidant activity [47]. Therefore, the antioxidant activity of compounds **1**–**3** may be attributed to their phenolic hydroxyl groups. Additionally, it was reported that antioxidant compounds produced by fungi could protect their hosts by enhancing tolerance to abiotic stresses [48]. Whether compounds **1–3** have similar effects deserves further exploration. In addition, these isolated compounds have been shown to have other bioactivities. For example, compound **1** was a common fungal metabolite with cytotoxic activities against the KB, MDA-MB-435 and MRC5 cell lines [49]. Compounds **2–3** were anthraquinone derivative and acted as an antimalarial and antiallergic agents [50, 51].

Symbiotic bacteria have occurred in some fungi, which affected fungi function and subsequent host-fungus interactions. However, only a few studies have explored their effects in insect-associated fungi [52, 53]. Here, we found the first insight into the bacterial symbionts in fungi associated with honeybee. The bacterial symbiont from honeybee has already been shown to protect the host by producing inhibitory compounds or providing nutrition [54, 55]. However, whether fungal-bacterial symbionts influence the growth of honeybee-associated fungi, and how they affect tripartite honeybee-fungus-bacterium mutualisms, remains to be explored further.

## Conclusions

Here, the diversity of the honeybee-associated fungi was investigated using both culture-dependent and culture-independent analysis methods. This study expands our knowledge of honeybee-associated fungi and further raises the pool of fungal species from *A. mellifera ligustica*. The results show that several of these fungi have antibacterial and antioxidant activities, among which the fungus *C. subaffine* MFFC22 was the most prominent. Furthermore, the antioxidant activities of *C. subaffine* MFFC22 can be attributed to the identified phenolic compounds. Collectively, the culturable honeybee-associated fungi provide insight into the widespread insect symbionts, which have great potential in finding the resource of novel species and antioxidants.

## Materials and methods

### Sample collection and microbial isolations

*A. mellifera ligustica* (including larvae, adults), honeycomb, and royal jelly were collected from the Institute of Apicultural Research, Anhui Agricultural University, Hefei, China (GPS: 31^◦^53ʹN, 117^◦^20ʹE) between April and July 2021. The honeybee larvae and adults starved for 24 h, and all samples were stored at 4 ℃. Initially, seven larvae, seven adults, and one gram honeycomb were placed separately into 10 mL sterile phosphate-buffered saline (PBS) buffer solution in an autoclaved 50 mL tube to obtain fungi from external isolation. Then, the same tube from the external was filled with 10 mL 75% ethanol for 2 min, followed by rinsing in sterilized 10 mL 1% bleach with 0.1% tween 20 for three times (30 s each). The supernatant was removed and replaced with 10 mL sterile PBS solution. Subsequently, sterile forceps were used to dissect samples of adult honeybee to get head, gut, and hypopharyngeal gland. Each body part was grounded in 10 mL sterile PBS. According to the earlier report, the honeybee larvae, one gram honeycomb, and one gram royal jelly were fully homogenized separately in 10 mL sterile PBS [56]. Then, the homogenates were diluted in a tenfold series (i.e., 10^−1^, 10^−2^, 10^−3^), and aliquots of 100 µL from each dilution were spread onto nine isolation media (Table 5). Pure colonies of fungi from the appropriate dilution were transferred into a new PDA medium and incubated aerobically at 28 ℃. All these isolated fungal strains were preserved on PDA slants at 4 ℃ until use. The fungi were used for freeze-drying preservation by a freeze-dryer (BTP-3ES; SP Scientific, USA) [57], and were stored at our institute.

**Table 5:** Culture media for the isolation of honeybee-associated fungi

Medium	Media Composition
ISP medium no. 2 (ISP 2)	Yeast extract 4.0 g, malt extract 10.0 g, glucose 4.0 g, agar 18.0 g, H_2_O 1000 mL
Potato dextrose agar (PDA)	Potato 200.0 g, glucose 20.0 g, agar 18.0 g, H_2_O 1000 mL
Lysogeny broth agar (LBA)	Yeast extract 5.0 g, NaCl 10.0 g, peptone 10.0 g, agar 18.0 g, H_2_O 1000 mL
Gauze’s modified medium no. 1 (GS)	Starch 20.0 g, KNO_3_ 1.0 g, K_2_HPO_4_·3H_2_O 0.5 g, MgSO_4_·7H_2_O 0.5 g, NaCl 0.2 g, FeSO_4_·7H_2_O 0.01 g, agar 18 g, H_2_O 1000 mL
Malt extract agar (MEA)	Malt 20.0 g, sucrose 20.0 g, peptone 1.0 g, agar 18 g, H_2_O 1000 mL
humic acid-vitamin (HV)	Humic acid 1.0 g, KCl 1.71 g, Na_2_HPO_4_ 0.5 g, CaCO_3_ 0.02 g, MgSO_4_·7H_2_O 0.05 g, FeSO_4_·7H_2_O 0.01 g, HVMulti-Vitamins(HV Multi-Vitamins: thiamine 0.05 g,riboflavin 0.05 g, inose 0.05 g, pantothenic acid 0.05 g, of p-aminobenzoic acid 0.05 g, vitamin B_6_ 0.05 g, biotin 0.025 g, niacin 0.05 g, ddH_2_O 100 mL), agar 18 g, H_2_O 1000 mL
Glycerine-peptone agar	Chitin 5.33 g,MgSO_4_ 0.0244 g, K_2_HPO_4_ 0.767 g, KH_2_PO_4_ 0.367 g, FeSO_4_·7H_2_O 0.01 g, ZnSO_4_·7H_2_O 0.001 g, MnCl_2_·4H_2_O 0.001 g, agar 18.0 g, H_2_O 1000 mL
Czapek Dox agar (CDA)	Sucrose 30.0 g, NaNO_3_ 2.0 g, K_2_HPO_4_ 1.0 g, KCl 0.5 g, MgSO_4_·7H_2_O 0.5 g, FeSO_4_·7H_2_O 0.01 g, agar 18 g, H_2_O 1000 mL
Sabaurauds dextrose agar (SDA)	Glucose 40.0 g, peptone 10.0 g, agar 18 g, H_2_O 1000 mL

### DNA sequencing

All honeybee-associated fungi were identified by molecular techniques and morphological characteristics [10, 58, 59]. Each fungus was cultured in malt extract medium at 28 ± 0.5 ℃ for 7 days. Then the fungal genomic DNA was extracted using the Fast DNA Extraction Kit (Aidlab Biotechnologies Co., Ltd., Beijing, China) as claimed by the manufacturer's specification. The primers ITS1/ITS4 were used to amplify ITS based on the fungal genomic DNA. Additionally, 16S rRNA was amplified using the primers 27F/1492R by PCR from the fungal metagenome [10, 60]. The quality of PCR products was visualized on 1% agarose gel by electrophoresis. Each product was successfully amplified from a PCR for sequencing (Tsingke Biotechnology Co., Ltd., Beijing, China).

### Identification of fungi and bacterial symbionts

As mentioned before [42], all achieved sequences' affiliation returned from Tsingke Biotechnology Company recognized by the available data in BLAST from the National Center for Biotechnology Information (NCBI) database. Sequence alignment and Neighbor-joining phylogenetic analysis were performed using MEGA software version 5.0. Bootstrap analysis of tree construction on the strength of the sequences was habituated to judge the neighbor-joining information based on 1,000 replicates [61]. The obtained ITS sequences were deposited in the GenBank database under accession numbers OK184563-OK184606 and OK285068. The accession numbers of bacterial 16S rRNA sequences were OK147622-OK147645 and OK169608-OK169611.

### Culture-independent community analysis

The sample pretreatment was the same as those mentioned above to obtain the honeybee gut and honeycomb. Then, the total genome DNA of samples was extracted using the Fast DNA Extraction Kit, the concentration and purity of DNA were confirmed on 2% agarose gels. Each sample was performed in triplicate. ITS1 genes of regions were amplified using a specific primer (ITS5-1737F and ITS2-2043R) with the barcode. The reaction conditions were 98℃ for 1 min, followed by 30 cycles of 98℃ for 10 s, 50℃ for 30 s, and 72℃ for 30 s, finally 72℃ for 5 min. Mixture PCR products were purified using Qiagen Gel Extraction Kit (Qiagen, Germany). Sequencing library was generated using TruSeq® DNA PCR-Free Sample Preparation Kit (Illumina, USA) according to manufacturer's recommendations and index codes were added. The library quality was assessed on the Qubit@ 2.0 Fluorometer (Thermo Scientific) and Agilent Bioanalyzer 2100 system. Finally, the library was sequenced on an Illumina NovaSeq platform using 250 bp pairedend reads.

Raw reads were demultiplexed and quality-filtered using QIIME V1.9.1 [62]. Effective Tags were obtained by comparing with the reference Unite database and using UCHIME Algorithm to remove chimera sequences [63, 64]. Sequences with ≥ 97% similarity were assigned to the same operational taxonomic units (OTUs) by using the software Uparse V7.0.1001 [65]. Taxonomy of each representative sequence was assessed by using the MUSCLE software V3.8.31, and comparison against the Unite database based on blast algorithm [66]. Raw data is available from the NCBI Short Read Archive under accession number PRJNA817087 and PRJNA817099.

### Cultivation of fungi and preparation of culture extract

Each fungus was inoculated in PDA medium and incubated at 28 ± 0.5 ℃for 3–4 days. Then, the fresh mycelia of each fungus (9 plugs of 5 mm) were inoculated in a 250 mL Erlenmeyer flask containing 150 mL of ME liquid medium and incubated at 28 ± 0.5 ℃ for 7 days in a shaker rotating at 180 rpm. The culture was passed through four layers of cheesecloth to remove the fungal thallus, then the supernatant was exhaustively extracted three times with ethyl acetate (EtOAc, 1:1, v/v). The fungal crude extracts were concentrated in a vacuum to yield culture extract for further experimental use.

### Antimicrobial activities

The filter paper disc method was used to screen the antibacterial activity of crude extracts [67]. Three bacterial strains (*Escherichia coli* (ATCC8739), *Micrococcus tetragenus* (ATCC35098), and *Staphylococcus aureus* (ATCC6538) were selected for the test and cultivated on tryptic soy blood agar (TSBA) medium in 37 ℃. Next, sterile filter paper discs (5 mm in diameter) were added to 5 µL of the tested crude extracts, which were dissolved separately in acetone to get a concentration of 18 mg/mL. The filter paper disc treated with acetone alone and gentamicin sulfate was served as a negative and positive control, respectively. The plates were prepared in triplicate and were cultivated at 37 ℃ for 24–36 h. Finally, the diameters of inhibition zone diameter (in mm) were measured for evaluating antimicrobial activity.

### Antioxidant activities

The 2,2-diphenyl-1 picrylhydrazyl (DPPH) radical scavenging activity was conducted according to the previous method with some modifications [68]. 1 mL of each crude extract (1 mg/mL) mixed with 5 mL of DPPH solutions in methanol (20 μg/mL). The mixture was incubated at room temperature for 30 min in darkness, and the absorbance was measured at 517 nm using a spectrophotometer (UV-1601; Beijing Beifen-ruili Analytical Instrument Co., Ltd., China). Methanol and ascorbic acid (Vc) were used as negative and positive controls, respectively. Each sample was performed in triplicate. The radical scavenging activity of crude extracts in the DPPH was calculated as follows:

Scavenging rate (SR) (%) = (A_b_- A_s_) /A_b_*100%

where SR was the scavenging activity of the tested sample (%), A_b_ was the absorbance without sample, and A_s_ was the absorbance in the presence of the samples or a positive substance.

### Isolation of compounds from MFFC22

A total of 13 L of the culture broth of MFFC22 was filtered and extracted with EtOAc (3 × 13 L). The EtOAc phase was concentrated under reduced pressure to obtain crude extract (2 g). The crude extract was subjected to column chromatography (CC) using silica gel (100–200 mesh) column with a gradient of CH_2_Cl_2_/MeOH (100:0–100:16, v/v) to give six fractions (Fr1-Fr6). Compound **1** (15 mg) was crystallized from the CH_2_Cl_2_ solution from the Fr1 (CH_2_Cl_2_ /MeOH, 100:0, v/v). Fr1 was further fractionated on a silica gel column, eluting with (CH_2_Cl_2_/MeOH, 100:0, 100:1, v/v) to give compounds **2** (0.9 mg) and **3** (0.7 mg).

### Structural elucidation of metabolites

The structures of all compounds were primarily analyzed by High Resolution-Mass Spectrometry (HR-ESI–MS) and ^1^H/^13^C-nuclear magnetic resonance (NMR) spectroscopies. HR-ESI–MS spectra were measured on a TripeTOF 4600 mass analyzer (Bruker, USA). ^1^H/^13^C NMR data were acquired using Agilent DD2 600 Hz spectrometer (Agilent, USA) with tetramethylsilane (TMS) as an internal standard, and chemical shifts (*δ*) were reported as parts per million (ppm) values.

### Antibacterial and antioxidant activities of compounds

The raw data supporting the conclusions of this manuscript will be made available to the authors, without undue reservation, to any qualified researcher. Publicly available datasets were analyzed in this study. This data can be found here: The obtained ITS gene sequences were deposited in the GenBank database under accession numbers OK184563-OK184606 and OK285068. The obtained 16S rRNA gene sequences were deposited in the GenBank database under accession numbers OK147622-OK147645 and OK169608-OK169611. Data of high-throughput sequencing by paired-end Illumina technology of ITS 1 gene amplicons can be retrieved from the NCBI Short Read Archive under accession number PRJNA817087 and PRJNA817099.

## Supplementary Information

**Additional file 1: Table S1.** Taxonomic assignment of the OTUs obtained in the study and their relative abundance at each honeybee gut.

**Additional file 2: Table S2.** Taxonomic assignment of the OTUs obtained in the study and their relative abundance at each honeycomb.

## Data Availability

The raw data supporting the conclusions of this manuscript will be made available to the authors, without undue reservation, to any qualified researcher. Publicly available datasets were analyzed in this study. This data can be found here: The obtained ITS gene sequences were deposited in the GenBank database under accession numbers OK184563-OK184606 and OK285068. The obtained 16S rRNA gene sequences were deposited in the GenBank database under accession numbers OK147622-OK147645 and OK169608-OK169611. Data of high-throughput sequencing by paired-end Illumina technology of ITS 1 gene amplicons can be retrieved from the NCBI Short Read Archive under accession number PRJNA817087 and PRJNA817099.
