# Correction: Community composition, bacterial symbionts, antibacterial and antioxidant activities of honeybee-associated fungi

**DOI:** 10.1186/s12866-022-02740-6

**Published:** 2022-12-22

**Authors:** Pu Cui, Kun Kong, Yong Yao, Zhongdi Huang, Shuping Shi, Peng Liu, Yechen Huang, Naeem Abbas, Linsheng Yu, Yinglao Zhang

**Affiliations:** 1 School of Life Sciences, Anhui Agricultural University, Hefei, 230036 China; 2 School of Plant Protection, Anhui Agricultural University, Hefei, 230036 China; 3 Laboratory of Functions and Applications of Medicinal Plants, Guizhou Medical University, Guiyang, 550014 China

**Correction: BMC Microbiol 22, 168 (2022)**

**https://doi.org/10.1186/s12866-022-02580-4**

In the originally published version of this article [1], we were notified that the following typos have been introduced during production:

In the Table 3 section:

“*S.S.S.S.S.S.S.S.aureus*” should be “*S. aureus*”.

In the Materials and Methods section:

**Antibacterial and antioxidant activities of compounds**

“The raw data supporting the conclusions of this manuscript will be made available to the authors, without undue reservation, to any qualified researcher. Publicly available datasets were analyzed in this study. This data can be found here: The obtained ITS gene sequences were deposited in the GenBank database under accession numbers OK184563-OK184606 and OK285068. The obtained 16S rRNA gene sequences were deposited in the GenBank database under accession numbers OK147622-OK147645 and OK169608-OK169611. Data of high-throughput sequencing by paired-end Illumina technology of ITS 1 gene amplicons can be retrieved from the NCBI Short Read Archive under accession number PRJNA817087 and PRJNA817099”. It should be as follows.

**Antibacterial and antioxidant activities of compounds**

The IZD of the compounds isolated from MFFC22 were determined using the method as previously described. *E. coli*, *M. tetragenus*, and *S. aureus* were used to assess the antibacterial activity of compounds. Compounds were dissolved separately in acetone to get a concentration of 6 mg/mL. Acetone and gentamicin sulfate served as negative and positive control, respectively. In addition, compound **1** at different concentrations was selected for the antioxidant experiment. Methanol and ascorbic acid were used as negative and positive controls, respectively. Each sample was performed in triplicate.
